# General Northern English. Exploring Regional Variation in the North of England With Machine Learning

**DOI:** 10.3389/frai.2020.00048

**Published:** 2020-07-15

**Authors:** Patrycja Strycharczuk, Manuel López-Ibáñez, Georgina Brown, Adrian Leemann

**Affiliations:** ^1^Department of Linguistics and English Language, University of Manchester, Manchester, United Kingdom; ^2^Alliance Manchester Business School, University of Manchester, Manchester, United Kingdom; ^3^Department of Linguistics and English Language, Lancaster University, Lancaster, United Kingdom; ^4^Center for the Study of Language and Society, University of Bern, Bern, Switzerland

**Keywords:** vowels, accent features, Northern English, random forests, feature selection, dialect leveling

## Abstract

In this paper, we present a novel computational approach to the analysis of accent variation. The case study is dialect leveling in the North of England, manifested as reduction of accent variation across the North and emergence of General Northern English (GNE), a pan-regional standard accent associated with middle-class speakers. We investigated this instance of dialect leveling using random forest classification, with audio data from a crowd-sourced corpus of 105 urban, mostly highly-educated speakers from five northern UK cities: Leeds, Liverpool, Manchester, Newcastle upon Tyne, and Sheffield. We trained random forest models to identify individual northern cities from a sample of other northern accents, based on first two formant measurements of full vowel systems. We tested the models using unseen data. We relied on undersampling, bagging (bootstrap aggregation) and leave-one-out cross-validation to address some challenges associated with the data set, such as unbalanced data and relatively small sample size. The accuracy of classification provides us with a measure of relative similarity between different pairs of cities, while calculating conditional feature importance allows us to identify which input features (which vowels and which formants) have the largest influence in the prediction. We do find a considerable degree of leveling, especially between Manchester, Leeds and Sheffield, although some differences persist. The features that contribute to these differences most systematically are typically not the ones discussed in previous dialect descriptions. We propose that the most systematic regional features are also not salient, and as such, they serve as sociolinguistic regional indicators. We supplement the random forest results with a more traditional variationist description of by-city vowel systems, and we use both sources of evidence to inform a description of the vowels of General Northern English.

## 1. Introduction

Dialect leveling is of central interest to sociolinguistics and dialectology. It is linked to dialect contact, and social mobility, and it is thought to arise through “avoidance or attrition of marked variants” (Trudgill, 1986). Such avoidance may lead to variation and change, in which regional variants are replaced with either standard or pan-regional ones. As such changes occur, regional variation is reduced. In the context of British English, there is robust evidence for leveling-type changes (Kerswill, 2003), and we may therefore ask how much regional variation still remains. Conceptually, this is a straightforward question, but empirically, it is not. In this work, we consider difficulties in quantifying the extent of regional variation in speech, and we propose some new methodological and computational solutions in this respect that rely on crowd-sourcing speech data, and quantifying variation with machine learning.

Our focus is on Northern British English, one of the main dialect groups in the UK. Northern British English can be defined in opposition to Southern British English, i.e., through the presence of linguistic features that are found in the North, but not in the South. These features may be syntactic (e.g., the use of the form “give it me” in Northern English), lexical (e.g., “spelk” as a regional variant for “splinter” in Newcastle), phonological or phonetic. We study phonological and phonetic features, understood as accent-specific realizations of specific vowels. Two features that provide a good demarcation between the North and the South in this respect is the presence of the trap-bath split and the foot-strut split in the South, but not in the North. Consequently, the bath vowel is shorter and relatively more front in the North, compared to the South, whereas the strut vowel is higher in the North compared to the South. This approach can lead us to consider Northern English to be a cluster of distinct but related varieties, which share a specific realization of bath and strut. However, some linguists use the term “General Northern English” (GNE) or “Standard Northern English” emerging as a more coherent variety spoken by certain speakers across the North, as a result of dialect leveling (Whiteside, 1992; Watt, 2002; Honeybone, 2007; Cardoso et al., 2019). GNE speakers can be expected to display typically northern features, like the northern bath and strut, but not other more narrowly defined northern features. For instance, Watt (2002) notes that traditional Tyneside realization of face and goat as centering diphthongs are avoided by middle-class Tyneside speakers. These speakers are generally shifting toward a pan-northern monophthongal variant, while Southern-standard-like closed diphthongal realizations are also present. Watt argues that many strongly localized accent features are eroding in Tyneside, under the influence of dialect contact. This, however, interacts with a development of a northern (or more narrowly in this case, north-eastern) identity that constrains dialect leveling such that the developing accents, although leveled, still sound distinctively northern. Tension between avoidance of certain regional features, and willing to signal one's northern identity is also noted by Wells (1982b), who says:

There are many educated northerners who would not be caught dead doing something so vulgar as to pronounce strut words with [ʊ], but who would feel it to be a denial of their identity as northerners to say bath words with anything other than short [a]. (Wells, 1982b, p.354)

To date, the following types of arguments have been proposed as evidence for General Northern English. One type of evidence is attitudinal, and it is expressed by speakers explicitly classifying their own accent as “northern,” as opposed to, for instance, “Geordie” (Newcastle) (Watt, 2002). Another type of evidence is gradual disappearance of certain regional features in favor of pan-regional forms, such as the avoidance of centring diphthongs for face and goat in Tyneside (Watt, 2002), and diphthongisation of the same vowels in York (Haddican et al., 2013). Thirdly, it has been observed that many northern accents participate in the same sound changes, which makes them more similar to one another. A striking example is goose-fronting, which is affecting multiple varieties of English world-wide, including Northern English accents, such as Bradford (Watt and Tillotson, 2001), York (Haddican et al., 2013), Manchester (Baranowski, 2017) and Carlisle (Jansen, 2019). While all this evidence points toward a degree of linguistic homogenization across the North, we may ask whether General Northern English can be considered a coherent variety, or whether it is still an umbrella term for a group of similar, but distinct accents.

We can phrase the same question in terms of classification: is it empirically justified to use labels such as “General Northern English” to describe the speech of some individuals, as opposed to more specific ones, like “middle-class Manchester English?” If geographically diverse northern speakers sound similar, and are thus difficult to localize within the North, we would take that as evidence for GNE. Implicit in this is the assumption that GNE is a middle-class accent. The issue of class is addressed in Cardoso et al. (2019), who investigate attitudes to accents in employment context, stratifying the sample for region and social class. They draw a distinction between GNE (standard, pan-regional and middle-class) vs. Leeds English (non-standard, regional and working-class). The same distinction can apply to Southern British English varieties, where Standard Southern British English is a non-localized standard, whereas Estuary English is an example of more localized, non-standard, working-class speech. The notion that relatively more standard accents are less regionally diverse is well-established in the dialectology of British English (Wells, 1982a). It is also supported by a long line of variationist work that consistently points to fewer regional features in middle-class speakers, compared to working-class speakers [relevant examples from the North of England, include Baranowski and Turton (2015) on Manchester English and Haddican et al. (2013) on York].

While there are indications of increasing homogeneity of middle class speech across the North of England, systematic evidence to support this intuition is limited. In this work, we investigate putative accent convergence in the North systematically, using an audio corpus of Northern English speech, and by using an explicit computational procedure. Traditional dialectology relies on the notion of accent features, and a comparison can be drawn between different accents by way of establishing that particular features are observed in accent A, as opposed to accent B. The more features are shared between two accents, the greater the similarity. This is a somewhat informal approach that essentially relies on expert intuitions about the relevant features for comparison. Such intuitions are eschewed in neighboring fields of computational linguistics and forensic linguistics, where more holistic approaches have been employed to automated accent recognition. Brown and Wormald (2017) propose a method for classifying accents out of a pre-specified pool, using acoustic information from all phones present in a speech sample. The method is based on a distance measure, computed using mel-frequency cepstral coefficients (MFCCs), and supplied to either a simple correlation calculation or a Support Vector Machine (as demonstrated in Brown, 2016). The models that are used within this process can also be supplied to a hierarchical cluster analysis to reveal the relative degrees of similarity that exists among a set of speakers' accents. Alternatively, we can apply a feature analysis to the speaker-specific accent models to estimate which phonemes are contributing most to distinguishing between different accent varieties. However, the method is only able to identify the relevant phones, but it does not provide an insight into how the specific phones differ between different accents.

In this work, we combine aspects of variationist and computational approaches to studying accent variation. We propose a new method for quantifying similarities between accents, based on random-forests based classification. Similarly to Brown and Wormald (2017), this approach allows to identify the features that are most reliable for distinguishing accents, and it provides a methodological solution for identifying key accent features in an explicit way. Unlike Brown and Wormald (2017), we rely on more traditional acoustic measurements, the first two vowel formants. Our approach has the advantage of being linguistically interpretable: we can not only find the vowel phonemes that distinguish different accents, but we can also describe the difference in linguistically meaningful terms, facilitating comparison with earlier descriptive work (e.g., vowel X is lowered in accent A, compared to accent B). This would not be possible if we used MFCCs, although the trade-off is including fewer phonemes (only vowels), and using less comprehensive acoustic information. The specific research questions for the analysis are:

1. To what extent can individual northern cities be systematically distinguished from the rest, based on vowel formant values?
2. Which vowels are the best predictors for each city?

In addition, we provide an up-to-date description of vowel systems in five cities: Leeds, Liverpool, Manchester, Newcastle upon Tyne and Sheffield, as represented in our speaker sample. The data from 105 speakers reading the same passage. In doing so, we follow the more traditional paradigm of plotting vowel spaces in a two dimensional acoustic space, defined by the first two vowel formants.

### 1.1. Selected Urban Varieties

**Figure 1:**
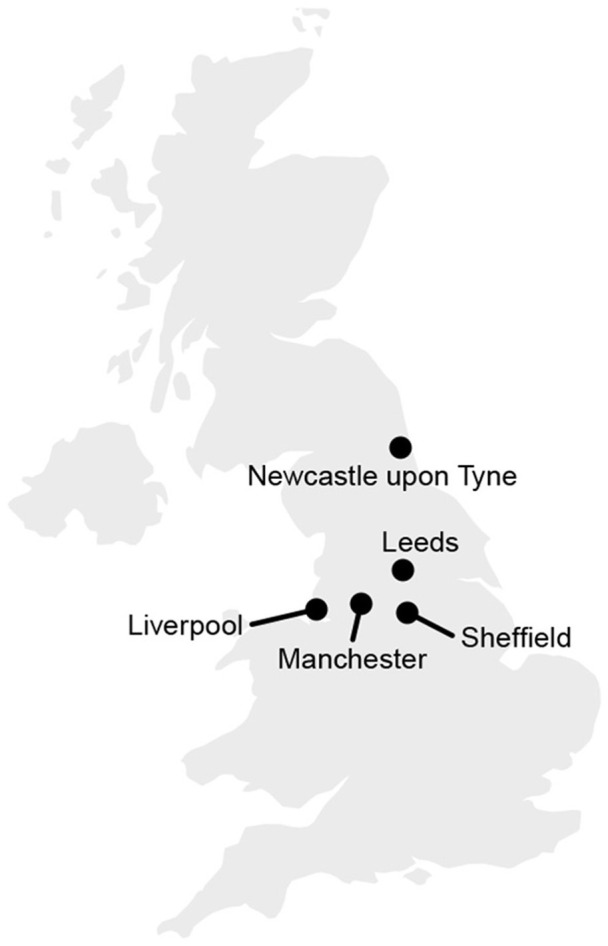
Geographical location of the five selected cities.

The accents we examine represent five urban localities in the North of England: Leeds, Liverpool, Manchester, Newcastle upon Tyne and Sheffield. Their relative location in the UK is presented in Figure 1. We chose to focus on urban varieties, because our approach relies on a categorical classification, and the different cities provide a robust way of grouping individual speakers geographically. Another motivation is that urban accents are likely to undergo leveling, due to increased speaker mobility and dialect contact. We selected the specific cities based on their shared characteristics: they are all relatively large urban centers in the North of England. An additional consideration was the availability of a sufficient number of speakers in the corpus we used (see section 2.1 for a description of the corpus). In our analysis, we focus on vowels only. This is because we can rely on a well-established method of quantifying differences between vowels, using formant measurements. For consonants, we would have to develop various types of phoneme-specific measurements, and it is less certain that these measurements would capture relevant variation equally well. Additional theoretical motivation for focusing on vowels comes from previous literature which posits that dialect leveling in British English tends to affect vowels more than consonants (Kerswill, 2003).

Below, we provide an overview of previous research on the vowels of the five selected cities. In the description, we use the parameters of variation in describing vowels of English, as developed by Wells (1982c). These are operationalized as lexical sets, selected based on phonemic distinctions in different varieties of English, and abbreviated as keywords. Wells's own description of regional accents are based on auditory transcription within the vowel quadrilateral framework that goes back to Jones (1917). Later works on varieties of English are often supported by acoustic measurements of the first two vowel formants. Recently, comparative dialect studies have been expanded to include articulatory information. We include data from such sources, although we are selective in our literature review, prioritizing sources that include comprehensive vowel descriptions and/or novel observations about specific vowel features. We include some of our own observations about the recordings acquired by the Dynamic Dialect project, where available. The Dynamic Dialects project provides ultrasound and lip video recordings of vowel keywords by speakers of 18 broadly defined accent areas (Lawson et al., 2018). This is a very useful point of reference for readers less familiar with northern accents, as the recordings are recent and freely available online at https://www.dynamicdialects.ac.uk/.

#### 1.1.1. Leeds

Leeds is a city in West Yorkshire, and its accent is described by Wells (1982b) as a prototypically northern. There is no foot-strut split, or trap-bath split. In addition, according to Wells, Leeds shows some traditional Yorkshire features, such as monophthongisation of face, goat and price. The realization of square in Leeds is transcribed as monophthongal by Wells, who also mentions the phonetic quality of north/thought as being very open. In addition, the happy vowel has a relatively lax quality.

#### 1.1.2. Sheffield

Sheffield is situated in South Yorkshire, and it shares a number of characteristics with Leeds. Among distinct characteristics of the Sheffield dialect, Wells (1982b) mentions a fronted onglide for mouth. Additional features of Sheffield English noted by Stoddart et al. (2014) include variable diphthongisation of fleece and goat, as well as variable fronting of onglide in goat. According to Stoddart et al., mouth can be monophthongal, and happy is lax.

A more recent description of the Sheffield accent is provided by Williams and Escudero (2014), who compare it to a Southern English system. Their averaged data for Sheffield speakers show diphthongal realization of face, goat and price, and there is no onglide fronting in mouth. These realizations are more similar to Southern English than to the traditional Yorkshire realizations, which is consistent with effects of dialect-leveling. However, statistical comparisons still show differences in the quality of these vowels between Sheffield and the southern accent. The general northern features, absence of [ʌ] and front lax realization of bath, are apparent in the data. In terms of more recent vowel changes, the 2014 Sheffield English data indicate the presence of goose-fronting, which is, however, less advanced than in the South.

Dynamic Dialects provides ultrasound recordings of two Sheffield speakers. These two speakers vary clearly in their production of face and goat. One of the speakers produces them as closing diphthongs, whereas the other speaker has more monophthongal variants. The price vowel is diphthongal for both. For both of them, the goose vowel appears somewhat fronted, in line with the data in Williams and Escudero (2014).

#### 1.1.3. Manchester

According to Wells (1982b), Manchester is very similar to Leeds in terms of vowels. However, in an updated description, Baranowski and Turton (2015) stress that face and goat are closing diphthongs in Manchester, and they do not have a monophthongal quality (this is in contrast to some Lancashire accents). Like other present-day varieties of English, Manchester shows fronting of goose, and to a lesser extent, goat. There is no goat-fronting before /l/. For goose-fronting, Baranowski and Turton also note an allophonic rule, which is furthermore sensitive to social variation. The goose vowel can be front before /l/ for working-class speakers, but not for middle-class speakers. Similarly, the realization of the strut vowel is socially stratified: middle-class speakers show relative lowering of strut. square and nurse are distinct. Baranowski and Turton (2015) also comments on the realization of happy and letter vowels. The happy vowel is relatively retracted and lowered, whereas letter is somewhat retracted. The letter vowel is reported in some sources to be lowered in Manchester (Beal, 2008). This aspect of Manchester speech is often stereotyped. It is not uncommon to see “Manchester” spelled as “Manchestaaa,” e.g., on social media, as a reference to the quality of the vowel. However, Turton and Ramsammy (2012) observe retraction rather than lowering in letter.

Data from a single speaker of Manchester English are available through Dynamic Dialects. Interestingly, this speaker has a lowered vowel in strut, which is distinct from foot, as observed for some Manchester speakers by Turton and Baranowski (2020). This speaker also has diphthongal face and goat (the onglide of goat is also fronted). Her happy vowel is relatively tense. In contrast, she shows the typically northern fronted production of bath.

#### 1.1.4. Liverpool

Compared to other northern accents, Liverpool is quite distinct, which is attributed to high migration levels into the city from a range of groups (Knowles, 1978). In terms of specific vowel features, Wells (1982b) mentions the merger between square and nurse, both of which are realized as a centralized vowel, rounded or unrounded. face and goat are diphthongal, and there is also a slight diphthongisation of fleece and goose. The vowel in happy is tense, unlike in Manchester and Yorkshire. In their study of Liverpool vowels, Ferragne and Pellegrino (2010) confirm this description, and they also note the phonetic proximity of *hod* and *hard* (lot and start), and between *hid* and *heard* (kit and nurse). According to Watson (2007), the price vowel can be monophthongal. Watson also notes optional goat-fronting. Furthermore, Cardoso (2015) observes a pattern of phonological variation in price and mouth in Liverpool, affecting the trajectories as a function of manner of articulation of the following consonant, and its voicing.

#### 1.1.5. Newcastle Upon Tyne

Traditional Newcastle English shows obvious differences from other northern accents. It is generally reported to display the northern strut and foot. Wells (1982b) notes that some bath and trap words can have a long [a:], unlike in most other Northern accents. He describes the Newcastle face and goat vowels as varying between monophthongs and centring diphthongs. fleece is said to be “strikingly diphthongal” in final position. The mouth vowel is variable, including some traditional [u:] realizations. Among the unstressed vowels, happy is relatively tense, whereas letter is said to have a particularly open quality.

Watt (1998) confirms that fleece and goose can be diphthongal in open syllables in Newcastle, whereas closed syllables invariably involve a monophthongal variant. Watt also documents extensively the variation in nurse, which includes a front rounded variant, as well as a strongly retracted one, and one that is close to Southern British English.

Ferragne and Pellegrino (2010) confirm aspects of this description, adding observations concerning front and close realization of nurse in Newcastle. They also comment on the variation in face and goat, including the monophthongal variants and centring diphthongs. Watt (2002) also includes a closing diphthong as a possible variant for face and goat, and he notes that such realizations are on the rise.

The Dynamic Dialects Newcastle speaker shows a monophthongal goat, and a centring diphthong for face, with a relatively lowered onglide. This speaker also shows fronting of the onglide in price. His happy is tense. Lowering in letter is not evident. nurse is relatively front, and the lip protrusion is evident in the video data. fleece is clearly monophthongal (that is in a closed syllable context).

## 2. Materials and Methods

### 2.1. Corpus

The data we use were extracted from the English Dialects App Corpus (EDAC, Leemann et al., 2018). The data are crowdsourced recordings of the passage “The Boy who Cried Wolf,” collected via a mobile phone app. At the time this paper was written, the corpus contained recordings from 3,500 speakers in the British Isles (including Republic of Ireland). Apart from donating the recording, the speakers identified their own accent by placing a pin on a map. This is an important aspect of the method: we do not use any additional criteria for defining an accent as belonging to a specific city, such as mobility, or family history. The speakers also provided demographic information, including age, gender, ethnicity, and level of education. A detailed description of the corpus is in Leemann et al. (2018).

An advantage of the EDAC corpus is that it uses controlled speech materials, which considerably reduces noise in comparing vowel realizations across different speakers and different groups of speakers. This enables us to work with a relatively smaller sample of speakers, compared to what we would have required if he used spontaneous speech. It also considerably reduces data processing time, obviating the need for manual orthographic transcription.

### 2.2. Speaker Sample Demographics

**Figure 2:**
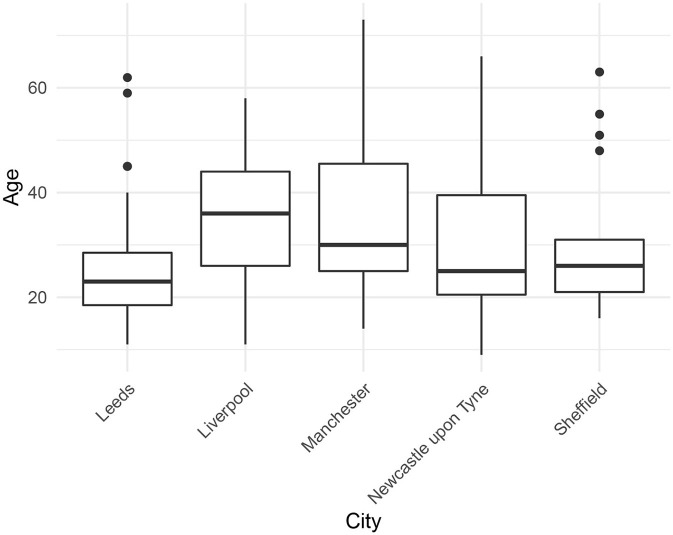
Speaker age by city.

We selected 105 speakers from the corpus, representing the five cities: Leeds (*N* =27), Liverpool (*N* =17), Manchester (*N* =23), Newcastle upon Tyne (*N* =19), and Sheffield (*N* =19). We chose recordings of sufficient quality, excluding those that were incomplete, had excessive background noise, multiple talkers present, etc. The mean speaker age was 31 years (*SD* =14). Figure 2 shows the distribution of speaker age by city. The individual cities are comparable in terms of age, although we note that the Leeds and Sheffield speakers were particularly young.

Fifty-nine percent of our speakers were female. As shown in Table 1, the balance of gender was similar across the different cities. In terms of ethnicity, the speakers were predominantly white (87.6%). 4.77% of speakers were Asian, 4.77% were mixed-race. The sample included one black speaker (from Sheffield), and five who did not identify with any of the ethnicity categories. The proportion of white speakers was roughly equal across the cities. The remaining ethnicities were not well-balanced. The by-city ethnicity data are summarized in Table 2.

**Table 1:** Summary of gender by city in our speaker sample.

**City**	**Gender**	**N**	**%**
Leeds	Female	16	59.26
Leeds	Male	11	47.4
Liverpool	Female	9	52.94
Liverpool	Male	8	47.06
Manchester	Female	14	68.7
Manchester	Male	9	39.13
Newcastle upon Tyne	Female	11	57.89
Newcastle upon Tyne	Male	8	42.11
Sheffield	Female	12	63.16
Sheffield	Male	7	36.84

**Table 2:** Summary of ethnicity by city in our speaker sample.

**City**	**Ethnicity**	**N**	**%**
Leeds	Asian	2	7.41
Leeds	Mixed	1	3.70
Leeds	Other	1	3.70
Leeds	White	23	85.19
Liverpool	Mixed	1	5.88
Liverpool	White	16	94.12
Manchester	Asian	2	8.70
Manchester	Mixed	1	4.35
Manchester	White	20	86.96
Newcastle upon Tyne	Asian	1	5.26
Newcastle upon Tyne	Other	1	5.26
Newcastle upon Tyne	White	17	89.47
Sheffield	Black	1	5.26
Sheffield	Mixed	2	153
Sheffield	White	16	84.21

As far as education is concerned, most speakers in a our sample (66.66%) had a higher education degree (BA or professional/vocational equivalent). 14.2% had been educated up to A-level, whereas 9.5% names GSCE as their highest level of education (this was specified as minimum five GSCEs grade A*–C)^1^. 9.5% of speakers had a lower qualification than that, including those that were under 16. The detailed by-city education data are summarized in Table 3. The individual cities are comparable in terms of speaker education, in that ca. 80% in each city had A-levels or a higher degree as their level of education. Education is the best proxy we have for social class, although we know that occupation may be a more reliable predictor (Baranowski and Turton, 2018). Based on the education data alone, we cannot conclude that all the speakers in our corpus are middle-class (in fact, that is almost certainly not the case), but we can expect that the corpus contains a substantial proportion of middle-class speakers.

**Table 3:** Summary of education by city in our speaker sample.

**City**	**Level of education**	**N**	**%**
Leeds	Higher	16	59.26
Leeds	A-level	4	14.81
Leeds	GSCE	4	14.81
Leeds	Lower than GCSE	1	3.70
Leeds	Under 16	1	3.70
Leeds	None	1	3.70
Liverpool	Higher	12	75.9
Liverpool	A-level	2	11.76
Liverpool	GSCE	1	5.88
Liverpool	Lower than GCSE	1	5.88
Liverpool	Under 16	1	5.88
Manchester	Higher	14	68.7
Manchester	A-level	5	21.74
Manchester	GSCE	1	4.35
Manchester	Lower than GCSE	1	4.35
Manchester	Under 16	1	4.35
Manchester	None	1	4.35
Newcastle upon Tyne	Higher	14	73.68
Newcastle upon Tyne	A-level	3	15.79
Newcastle upon Tyne	GSCE	1	5.26
Newcastle upon Tyne	Under 16	1	5.26
Sheffield	Higher	14	73.68
Sheffield	A-level	1	5.26
Sheffield	GSCE	3	15.79
Sheffield	Under 16	1	5.26

Summing up the demographic data, a typical speaker in our sample is an urban white woman in her 30s with a university degree. This speaker profile differs noticeably from the Non-Mobile Old Rural Male archetype traditionally associated with the dialectological paradigm. However, for the purpose of researching GNE, the sample is well-suited, especially in its education characteristics, as we can expect speakers with higher levels of education to display more standard features and fewer regional ones.

### 2.3. Materials

As previously mentioned in section 2.1, the speakers read the story of “The Boy Who Cried Wolf.” This is a very short text (216 words), which nonetheless contains all English vowels (based on standard descriptions), and so it is appropriate material for investigating English vowels, according to Deterding (1997). We selected one word representing each keyword, as listed in Table 4. In selecting the words, we tried to choose monosyllabic words, but it was not always possible. We could not find consistent selection criteria in terms of segmental and prosodic context, so the set is not well-controlled for in that regard. We keep those limitations in mind when analyzing the results. We acknowledge that we could potentially observe more regional variation related to allophonic alternations if we could vary the segmental and prosodic context systematically. All the keywords, bar one, are based on Wells (1982c). As an additional keyword, we included fool. This keyword was chosen to capture the fact that for most younger speakers across many varieties of English, a back [u:] vowel can only occur before a coda /l/ (as in *fool*), whereas in other contexts, the goose vowel is fronted to [ʉ:] or [y] (Strycharczuk and Scobbie, 2017a). Furthermore, this allophonic variation is sensitive to regional and social variation, such that /u:/-fronting before an /l/ is attested for some speakers in Manchester (Baranowski and Turton, 2015) and Liverpool (Hughes et al., 2012).

**Table 4:** Words selected for measurement with corresponding keywords.

**Item**	**Keyword**
*feast*	fleece
*fist*	kit
*zoo*	goose
*plan*	trap
*afternoon*	bath
*dark*	start
*thought*	thought
*hot*	lot
*foot*	foot
*duck*	strut
*third*	nurse
*shepherd*	dress
*fool*	fool
*short*	north
*safety*	happy
*safety*	face
*homes*	goat
*shouting*	mouth
*time*	price
*boy*	choice
*fear*	near
*air*	square
*however*	lettER

### 2.4. Data Processing

The selected recordings were forced-aligned using an HTK-based forced aligner developed in house. The vowel boundaries were then manually checked by two Undergraduate Research Assistants for all the selected items, listed in Table 4. We measured the first two formants automatically, using Praat. For monophthongs, we measured the formants at midpoint. For diphthongs, we used the onglide and offglide as selected time points, defined as 20 and 80% of the vowel duration respectively. The monophthong-diphthong distinction can differ across different accents. We considered all Standard Southern British English (SSBE) diphthongs as potential diphthongs, and measured them at two points, i.e., choice, face, goat, mouth, near, price, and square. This is based purely on convention, and it should not be taken as a statement about the dynamic characteristic of any vowel. The convention is not perfect. For instance, square is often monophthongal, whereas fleece and goose can be diphthongized. However, making principled decisions about the classification of each vowel in dynamic terms would require a separate in-depth analysis, and as such, it is beyond the scope of our investigation. Our primary interest is in comparing vowels across different accents, and we assume that measuring vowels at consistent time points for different accents should be sufficient to pick out the relevant cross-accent differences in vowel quality.

We used the Linear Predictive Coding algorithm in Praat to extract the measurements, based on 5 formants, 25 ms Gaussian window and 50 Hz pre-emphasis. For male speakers, the maximum formant was set at 5 kHz, whereas for females speakers, it was 5.5 kHz. All the measurements were checked by PS, and hand-corrected wherever tracking errors were spotted. Although manual corrections affect the reproducibility of our measurements, they were deemed necessary, because we rely on one vowel measurement per keyword per speaker, which makes the analysis sensitive to outliers. Ca. 10% of the measurements were hand-corrected.

### 2.5. Analysis

The formant data were *z*-scored within speaker (a modification of Lobanov, 1971). We used the normalized vowel formant measurements as the input to the random-forest based classification. The purpose of the analysis was to establish how individual urban accents differ from the ones representing other cities. This allows us to assess the distinctness of each accent, and to identify the specific vowel features that set individual northern cities apart. Accuracy of the models was evaluated using leave-one-out cross-validation. We illustrate the procedure using Manchester as an example. For each speaker, we constructed a training dataset by removing this speaker from the data. We then created a bootstrapped sample, with equal number of Manchester and non-Manchester speakers, using the remaining data. We under-sampled the majority class to create a balanced sample. We trained a random forest model on this dataset and tested its accuracy by predicting whether the left-out speaker was from Manchester or not. This procedure was repeated 100 times per speaker, resampling the bootstrapped sample each time, and averaging the predictions, a procedure known as bootstrapped aggregation (bagging, Breiman, 1996). We used the default settings of the current version (1.3–3) of the party package, which are the settings suggested for the construction of unbiased conditional random forests by Strobl et al. (2007). In particular, we used mtry=5, where mtry is defined as the “number of input variables randomly sampled as candidates at each node” (Hothorn et al., 2020), and minicriterion=0, where minicriterion is a parameter that controls the depth of the trees (minicriterion=0 grows trees of maximal depth). We further tested different settings for mtry, checking for potential improvements in accuracy, depending on the settings. We find no overall improvement in accuracy for higher values of mtry beyond 5.

**Figure 3:**
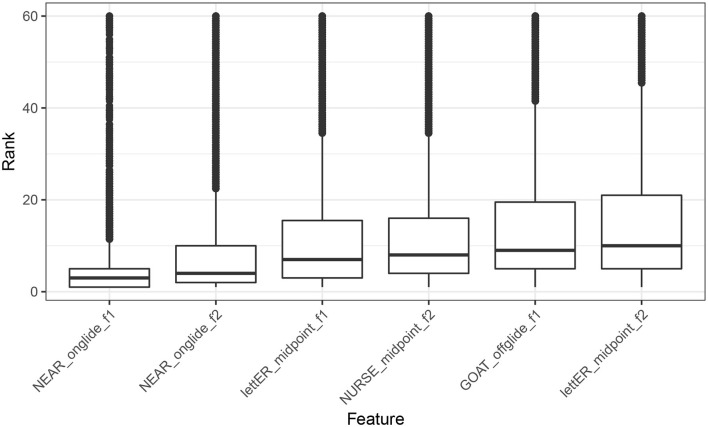
Distribution of feature ranking across all the models for Manchester.

From each model, we extracted conditional variable importance (Strobl et al., 2008), and ranked the features, according to their relative contribution. We then analyzed the distribution of feature ranking visually, in order to determine which vowels are most consistently used to identify Manchester. The distributions of top ten highest ranking features for Manchester are visualized in Figure 3. As we can see, the F1 of near, measured at onglide ranks the highest, followed by onglide F2 for the same vowel. Note that “F1” and “F2” refer to vowel formants here and throughout the paper.

The methodological decisions in setting up the analysis were made to address some of the challenges introduced by the nature of our data. The use of random forests was motivated by the possibility of calculating conditional feature importance, which allows us to identify which input features have the largest influence in the prediction, i.e., which acoustic properties of which vowels set individual cities apart. With the same aim in mind, we set the dependent variable as binary, i.e. Manchester vs. other northern urban accents, Liverpool vs. other northern urban accents, etc. This, however, creates an unbalanced sample, as in each case, the negative category (data from other cities) is about four times bigger than the data from the target city. In order to address this and create balanced data, we used under-sampling. Since under-sampling excludes useful data from the resulting data sample, we used bagging to consider many possible balanced data samples. Given that the data set is relatively small, we were not in a position to split the data into a training test and test set based on a 25–75% split, as is common in random forest modeling. We used leave-one-out cross-validation instead, so we could evaluate accuracy on unseen data, while maximizing the amount of training data.

**Figure 4:**
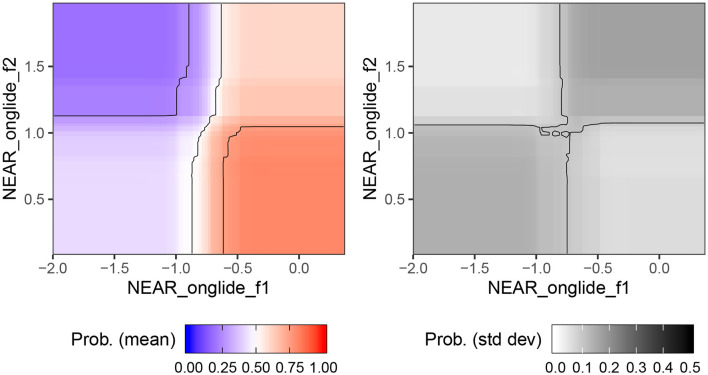
Certainty of the random forests predicting Manchester based on F1 and F2 of near measured at onglide.

In order to get more insight into the effect of individual predictors, we used the same bagging process as above (fitting random forest models on a bootstrapped balanced sample that under-samples the majority class), but using only the two features that consistently ranked as most important. We did this without the leave-one-out procedure, and used 1,000 bootstrapped samples per city. We then computed forest predictions for the whole range of values of these features. The output is a heatmap, as visualized in Figure 4. The left panel shows the mean, over the 1,000 random forest models, of the probability of predicting Manchester. This is visualized using color scale, where highest certainty of identifying Manchester is associated with relatively darker shade of red. As we can see, the likelihood of an accent being classified as Manchester increases for higher F1 and lower F2 values of the near onglide. Based on established correlations between formant values and tongue height and tongue position, we can interpret this result as follows: Manchester accents are associated with a lowered and centralized onglide for near. The right panel of Figure 4 shows the standard deviations for the conditional class probabilities.

We also used the formant measurements to generate by-city vowel plots, and we use those for qualitative data analysis. The vowel plots are in section 3.2, and they show by-vowel median formant values for each city. In order to improve the legibility of the plots, we plot tense monophthongs, lax monophthongs and diphthongs separately. We consider bath to be lax, based on previous descriptions in northern varieties (see section 1.1). Otherwise, the grouping is based on the same convention as discussed in section 2.4 above.

The data were analyzed in R (R Development Core Team, 2016). The random forests were fitted using the party package 1.3–3 (Hothorn et al., 2006). The vowel plots were generated using modified code originally written by M. Winn (http://www.mattwinn.com/tools.html).

## 3. Results

### 3.1. Random-Forest Results

#### 3.1.1. Accuracy

We measure accuracy as the number of correct classifications, using the leave-one-out approach (see section 2.5), as a percentage of the number of trials. Table 5 provides the accuracy values for each city, along with sensitivity (true positives), and specificity (true negatives) values. Overall, the frequency of correct classification was relatively high for Liverpool (82%) and Newcastle (71%). For the remaining cities, it was lower with 67% for Leeds and 63% for Manchester. For Sheffield, the classification was close to random with 55% accuracy.

**Table 5:** By-city classification accuracy.

	**Leeds**	**Liverpool**	**Manchester**	**Newcastle**	**Sheffield**
Accuracy	67	82	63	71	55
Sensitivity	74	86	72	73	60
Specificity	65	81	60	71	54

If different northern English dialects are becoming more alike, this is predicted to lower the prediction accuracy for the classification models. The overall accuracy results suggest a certain degree of dialect leveling, especially affecting Manchester, Sheffield and Leeds. This is further supported by the accuracy figures broken down for pairs of cities. Table 6 shows the accuracy figures for each set of forests (forests trained on Leeds as positive category, Manchester as positive category, etc.), in classifying speakers from each remaining city. In this case, correct classification is always negative. This summary confirms that Liverpool and Newcastle are generally well-discriminated from the remaining cities. In contrast, Leeds and Sheffield are highly confusable. Forests trained on Leeds data are more likely than not to classify Sheffield speakers as coming from Leeds. The same situation occurs for models trained on Sheffield: they tend to classify Leeds speakers as coming from Sheffield. There is also a degree of confusability between Leeds and Manchester: classification is close to 50% for this pair of cities, although it is marginally better than random.

**Table 6:** Classification accuracy for pairs of cities.

**Predicted city: Leeds**			**Predicted city: Sheffield**			**Predicted city: Manchester**		
**(correct if predicts not Leeds)**			**(correct if predicts not Sheffield)**			**(correct if predicts not Manchester)**		
**True city**	**% Correct**	**% Incorrect**	**True city**	**% Correct**	**% Incorrect**	**True city**	**% Correct**	**% Incorrect**
Liverpool	93	07	Liverpool	71	29	Newcastle	64	36
Newcastle	73	27	Manchester	57	43	Liverpool	63	37
Manchester	53	47	Newcastle	54	46	Sheffield	62	38
Sheffield	47	53	Leeds	40	60	Leeds	55	45
**Predicted city: Liverpool**			**Predicted city: Newcastle upon Tyne**					
**(correct if predicts not Liverpool)**			**(correct if predicts not Newcastle)**					
**True city**	**% Correct**	**% Incorrect**	**True city**	**% Correct**	**% Incorrect**			
Leeds	86	14	Liverpool	73	27			
Sheffield	83	17	Leeds	75	25			
Newcastle	79	21	Manchester	71	29			
Manchester	76	24	Sheffield	63	37			

#### 3.1.2. Distinguishing Features

The features with the highest median ranking of feature importance for each forest are listed in **Table 8**. The table also provides the direction of prediction for each city, which is based on the heatmaps. We focus on two features for each city, based on the observation that there was typically a large difference in median ranking between the two top features and the rest. This suggests that most forests tend to rely most heavily on the same features. We confirmed this by refitting the forests based on top two features for each city only, and analyzing the resulting accuracy. As can be seen in Table 7, the accuracy only degrades slightly. We find the biggest drop in accuracy for Liverpool, but the accuracy is nonetheless still high at 73%. For Sheffield, we find an improvement in accuracy, which suggests that having more features leads to overfitting. These results should not be taken to mean that other features do not contribute to the prediction. Since some vowel formants are generally correlated with each other (e.g., thought and north, diphthongs offglides), we expect that a reasonable degree of accuracy could also be achieved based on different feature combinations, and this is confirmed by exploratory further modeling we have done. Dealing with highly correlated features is one of the strengths of conditional random forests (Strobl et al., 2007), and the known existence of correlations was precisely one of the reasons for choosing this method.

**Table 7:** By-city classification accuracy based on top two features only.

	**Leeds**	**Liverpool**	**Manchester**	**Newcastle**	**Sheffield**
Accuracy	64	73	63	65	66
Sensitivity	64	72	64	66	58
Specificity	64	73	63	65	67

According to forest prediction, the kit vowel is raised in Leeds, while north is lowered. For Sheffield, the top ranking features are a particularly retracted realization of fool and raised onglide of near. The onglide of near is also the most prominent feature for classifying Manchester: in Manchester, the onglide is relatively lowered and centralized.

Based on the random forests, the most systematic features of Liverpool accent are a lowered letter vowel and a fronted fool. Newcastle has a considerably lowered strut vowel. The second high-ranking feature for Newcastle is the offglide of price, which is fronted, compared to other cities.

### 3.2. By-City Vowel Systems

In this section, we present qualitative comparisons of median by-city vowel systems, focusing on the features previously discussed in the literature, summarized in section 1.1. The reader is reminded that the vowel plots are based on one word per vowel, and the segmental context was not controlled for between vowels, but it was consistent between cities (see Table 4 for the items we used). Therefore, distances between any two vowels within a city might be skewed, but vowels are comparable between cities. Our description is based on medians, and we do not take variance into account at this stage. Therefore, any observed differences should be taken with caution.

**Figure 5:**
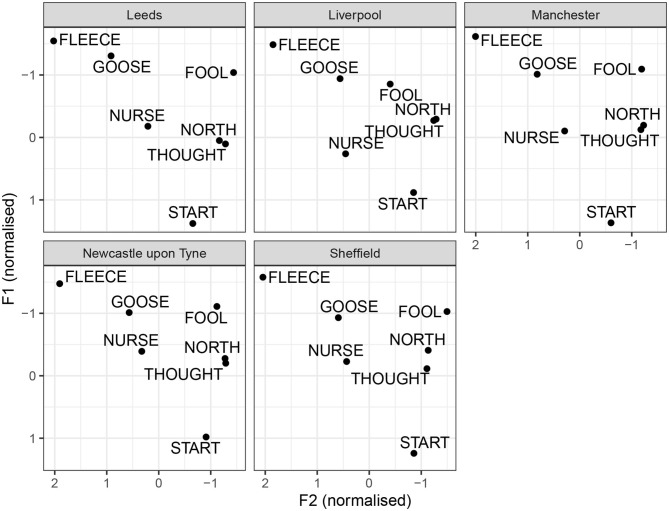
By-city tense vowel systems.

We begin with tense monophthongs, illustrated in Figure 5. For tense monophthongs, the results seem broadly consistent with previous descriptions. goose is not a back vowel for any of the accents. However, the degree of goose-fronting varies between cities. It is most advanced in Leeds and Manchester, followed by Liverpool, Sheffield and Newcastle. Furthermore, goose is somewhat higher in Leeds, compared to other cities. Furthermore, all cities show considerably more fronting in goose than fool. However, there are regional differences in the degree of fool-fronting. In Sheffield and Leeds, fool is back. The similarity between Sheffield and Leeds in this respect may be one of the factors contributing to the confusability between the two cities, seeing how fool retraction is one of the main features of Sheffield. In Liverpool, there is a considerable degree of fool-fronting, consistent with what is identified by the random forest analysis. Manchester and Newcastle show in-between median degrees of fool-fronting, but the vowel can still be considered back. Another vowel showing some variation is nurse. It is considerably lowered in Liverpool, compared to other cities. In Newcastle, the median nurse realization is mid and front-centralized. It resembles most other cities (and SSBE), as opposed to fronted and retracted variants noted in Tyneside (see section 1.1.5). The thought vowel is somewhat lowered in Leeds. Although the difference is subtle, thought-lowering is picked out by the random forest analysis as a distinguishing feature for Leeds. This is also consistent with Wells's 1982b description of the open quality of thought in Leeds.

**Figure 6:**
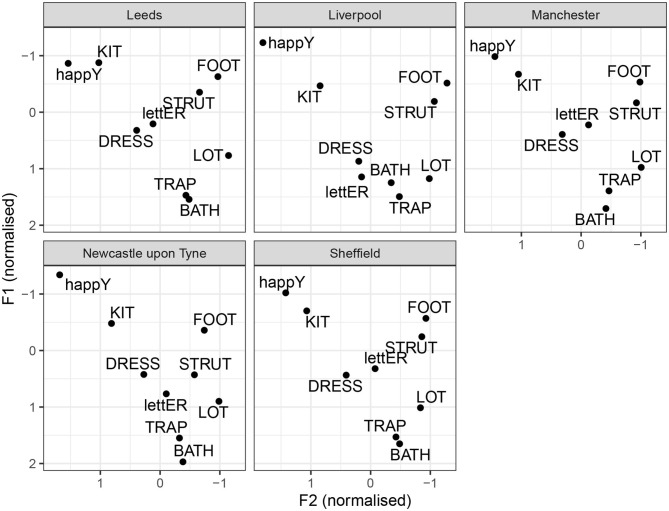
By-city lax vowel systems.

The by-city lax vowel systems are illustrated in Figure 6. Once again, these are median values without measures of dispersion. Regional differences can be noted in the F1 of happy. The vowel is higher in Liverpool and Newcastle, compared to Leeds, Sheffield, and Manchester. This is consistent with previous descriptions about the regional distribution of happy-tensing, as present consistently in Liverpool and Newcastle, but not in Manchester or Yorkshire. Nevertheless, happy is higher than kit for all cities, except Leeds, which is however, due to kit being exceptionally raised in Leeds (same as in Table 8). There does not seem to much evidence for foot-fronting in any of the cities, unlike in SSBE (Hawkins and Midgley, 2005; Strycharczuk and Scobbie, 2017b). Note that, in the present data, foot tends to have similar degree of acoustic backness to lot. There seems to be some slight foot-fronting in Newcastle, whereas in Liverpool, the foot vowel is the most retracted. The strut vowel is lower than foot for all cities, and it is especially low in Newcastle, where strut is clearly distinct from foot. The trap and bath vowels show some regional variation in height, but generally bath is as front as trap for all cities. The dress vowel is somewhat lowered in Liverpool, compared to other locations. The letter vowel is very similar in Leeds, Sheffield, and Manchester, but relatively more open in Liverpool and Newcastle.

**Table 8:** Highest ranked features for each city.

**City**	**Feature**	**Direction**	**Articulatory interpretation**
Leeds	kit midpoint F1	lower	vowel is raised
	north midpoint F1	higher	vowel is lowered
Sheffield	fool midpoint F2	lower	vowel is retracted
	near onglide F1	lower	onglide is raised
Manchester	near onglide F1	higher	onglide is lowered
	near onglide F2	lower	onglide is retracted
Liverpool	letter midpoint F1	higher	vowel is lowered
	fool midpoint F2	higher	vowel is fronted
Newcastle upon Tyne	strut midpoint F1	higher	vowel is lowered
	price offglide F2	higher	offglide is fronted

In comparison to previous descriptions, our results largely confirm that the reports about the regional distribution of happy tensing. They also confirm that, across the North of England, the bath vowel patterns with trap. The lowering of letter in Newcastle is consistent with the description by Wells (1982b). However, we also find letter lowering in Liverpool, where it had not been noted. Conversely, the Manchester letter vowel is not lowered, contra the stereotype. The dress vowel also seems lowered, as well as centered in Liverpool. Perhaps most strikingly, for all cities, and especially in Newcastle, we find some strut lowering, relative to foot.

**Figure 7:**
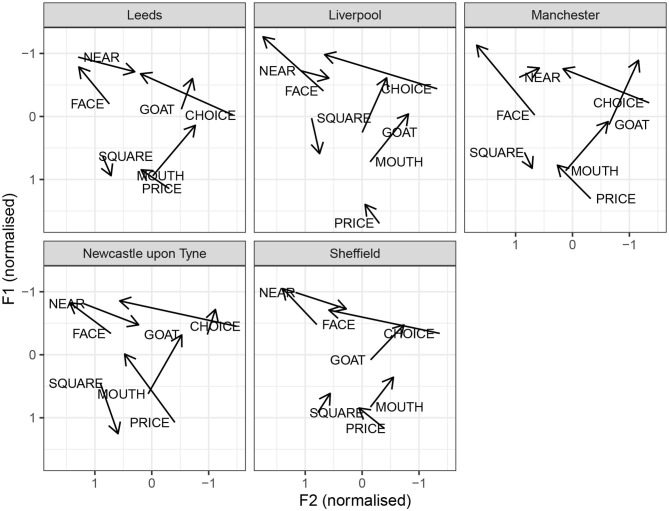
By-city diphthong vowel systems.

Figure 7 illustrates the diphthongs systems for the individual cities. Impressionistically, the diphthongs appear to show more regional variation than monophthongs. face is a closing diphthong for all cities. The median values do not include monophthongal varieties, as reported for Yorkshire, or centring diphthongs, as reported for Newcastle. In Manchester and Liverpool, face seems to be more diphthongal, compared to other cities. A similar generalization can be made for goat: it is a closing diphthong overall, and it is relatively wider in Manchester and Liverpool. Furthermore, there is regional variation with respect to the onglide of goat. In Liverpool, there is quite clear goat-fronting. The offglide of goat is also more front in Leeds and Sheffield, compared to Manchester and Newcastle. The price vowel has a relatively back and low onglide for all cities. The offglide, however, differs by city. In Liverpool, price is relatively monophthongal, which, however, is likely due to the segmental context, since was followed by a nasal (*time*), and price monophthongisation before nasal is noted for Liverpool by Knowles (1978). In Newcastle, price is a very wide diphthong. The remaining cities have an in-between, but clearly diphthongal variant. The mouth vowel is relatively stable across the cities. The near vowel is a centring diphthong in Leeds, Sheffield, and Newcastle. In Liverpool and especially Manchester, it is considerably more monophthongal. In Liverpool, it still seems to have a centring, if a short, trajectory. In Manchester, the offglide is somewhat higher than the onglide, but there is very little movement overall. The square vowel is quite clearly diphthongal in Newcastle, with a surprisingly low offglide. In comparison, other cities have a more monophthongal variant. In Liverpool, the square vowel is relatively raised, overlapping in the formant range with nurse, consistent with previous reports of a nurse-square merger.

## 4. Discussion

The main hypothesis underlying this research is that a large group of speakers in the North of England are converging to a pan-regional standard, and therefore, they cannot be localized further within the North, based on their vowel system. We investigated this by quantifying the success of random forest models trained to differentiate selected Northern English urban accents from a mixed pool of other Northern English accents. Overall, we find that two northern urban accents, Liverpool and Newcastle, remain quite distinct, and therefore they pose few challenges to classification, whereas we do observe a degree of classification uncertainty between Manchester and Leeds accents, and even more so between Leeds and Sheffield accents. From previous descriptions, we would expect that Manchester, Leeds and Sheffield accents are more similar to one another than Newcastle or Leeds. However, our current results allow us to quantify this observation with more precision: while some speakers from these cities can be reliably classified in terms of their accents, in about half of the cases, Leeds and Sheffield speakers in our data are mutually misclassified. Similarly, the classification accuracy for the Manchester—Leeds pair approaches random.

Similarities between the vowel systems of Leeds, Sheffield, and Manchester are further confirmed by the median vowel measurements for each city, as shown in Figures 5–7. For example, for all three cities, the typical happy realization is tense, goose is fronted, whereas fool is retracted, and face, price, and choice are all closing diphthongs. All these features are, broadly, also observed in Southern British English, and their robust presence in our data can be taken as a sign of dialect leveling in the North toward a more general British Standard. However, some general northern features prevail, including fronted realization of bath (consistent with no bath-trap split) and a raised strut vowel. The three accents also share a monophthongised realization of square, which is considered a general northern feature by Honeybone (2007).

The realization of strut warrants further comment: while the vowel is relatively raised for all cities (less so for Newcastle), it is not identical to foot. This is different from SSBE, but also different from traditional descriptions of Northern English that report no distinction between foot and strut as one of the identifying features of Northern British English. We must be careful in the phonological interpretation of the phonetic difference we observe. The measurements are not based on a minimal pair, so we cannot be certain that the observed difference in medians is due to a phonemic split between strut and foot. However, it seems unlikely that a difference of this magnitude would be due to phonetic coarticulation alone. The test items we used were *duck* and *foot*, and there is no reason to expect a strong F1 raising effect in the case of *duck*. We had examined the realization of foot and strut more systematically in Strycharczuk et al. (2019), using the same corpus, but including more tokens. We found that about 25% of speakers in the corpus have a phonemic split between foot and strut, while many more have a small but systematic phonetic distinction in the same direction. Thus, the most accurate characterization of the strut vowel in the North of England, according to our data, is that the vowel is considerably raised compared to Southern British English [ʌ], but the quality is not necessarily identical to foot. A similar observation is made by Turton and Baranowski (2020), based on socially stratified sample of speakers from Manchester. Turton and Baranowski show that the degree of strut systematically correlates with social class, with more lowering present in middle-class speakers, compared to working class.

We would argue that the vowel systems for Leeds, Sheffield, and Manchester, as presented in our paper, are all representative of pan-regional General Northern English. At the same time, however, this variety is not a monolith. Some systematic differences between these cities are present in our data. One striking example is the near vowel in Manchester, which has a distinct realization, with a lowered and centralized onglide. Further analysis of sample distribution of F1 and F2 in the onglide of near reveals the presence of even more extremely centralized variants, and these are confined to Manchester. For Leeds, kit raising is very distinct, and in this case, we see relatively little overlap in F1 values for kit between Leeds and other cities.

A key outcome of our study is that the features we find to be of most systematic importance in distinguishing individual northern accents are typically not traditional accent features. Among the features listed in Table 8 only one, north lowering in Leeds is mentioned in a previous description, Wells (1982b), as characteristic of that city. In a way, this is in line with the prediction that dialect-leveling targets salient regional features (Trudgill, 1986; Kerswill, 2003). It is then also expected that less salient regional features may be resistant to leveling. We also believe there is an additional reason why some lesser described features emerge as most important for the classification. To understand this, we need to consider that the success of machine-based classification is facilitated by features that show high-across city and low within-city variation. If the sample from any particular city mostly contains fairly standard speakers, and these speakers make up the most of the training data, the model might not be successful in classifying a speaker who has some very distinct regional features, but who is thereby also very different from the other speakers in the same sample. In contrast, the machine learner performs better with features that are highly consistent, even if the requisite phonetic differences are small. It may also ignore some features that are not consistent within the sample. This is different from a human listener, who is more likely to pay attention to features that are striking, even if such features are less systematic. Translating this distinction into the Labovian paradigm of indicators, markers and stereotypes (Labov, 1972), machine learners will be highly sensitive to indicators, features that systematically distinguish dialects, but that are not the subject of sociolinguistic awareness. It is the absence of sociolinguistic awareness that makes such features systematic within a dialect. Human listeners, on the other hand, are more likely to pick up on markers and stereotypes, by the very definition of markers and stereotypes. This also has consequences in production: speakers are more likely to avoid (some) markers or stereotypes when trying to sound standard.

**Figure 8:**
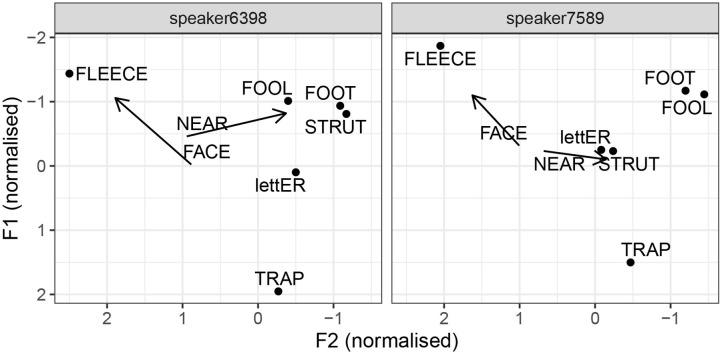
Selected vowels by two Manchester speakers.

This point is illustrated by two speakers, each of whom scored 100% accuracy across 100 simulations set up to identify Manchester. This means that 100 models based on different samples, all of which excluded the speaker in question, correctly classified that speaker as coming from Manchester. Figure 8 shows the formant values for selected vowels, as pronounced by the two speakers. To a linguist, two differences between these two speakers immediately stand out. Speaker 6398 shows has fool-fronting, a feature we find in Liverpool English, and which has also been reported in Manchester working class speech. In contrast, speaker 7589 has a retracted fool vowel. The two speakers also differ with respect to the foot–strut contrast: speaker 7589 has a very clear contrast, and the magnitude of the distance seems consistent with a phonological split. Speaker 6398 does not seem to have a difference between foot and strut, or if there is a difference, it is phonetically marginal. Based on these features, speaker 7589 seems more standard, and in fact, closer to the southern standard, given her pronunciation of strut. Speaker 6398, on the other hand, shows clear northern features, including some non-standard ones. However, they both have a lowered and centralized onglide for near. The fact that speaker 7589 incorporates this vowel into an otherwise very standard system corroborates our proposal that lowered and centralized near is an indicator of Manchester speech. This vowel is pronounced differently in Sheffield and Leeds, where the onglide is very close to the offglide of face (see Figure 7).

The differences between the two speakers in Figure 8 and the ways they differ from the Manchester median in Figures 5–7 also bring up an important point about individual variation. We may ask whether these two speakers speak GNE. Are they examples of individual variation within GNE, or do they represent a degree of variation from the standard? If we define GNE as a set of vowel target realizations, then we might be inclined to say the two Manchester speakers are not representative of this variety, or even the Manchester version of it. However, under such a narrow definition, we might find that very few individuals do, in fact, speak GNE. Alternatively, we can also define GNE not by the kind of features we find in the majority of middle-class Northern English speakers, but also by the kind of features we do *not* find. What we do not find is certain marked regional variants, which we can suspect, are perceived by speakers as markers of social class, or lower social prestige. Examples of these include traditional Yorkshire features, such as monophthongised face, or a lowered letter in Manchester. As another possibility, we can define GNE in terms of ranges of possible variation that are set differently for different vowels. For example, there may be a degree of variation possible for the strut vowel, such that raised realization as well as some degree of lowering can both be considered GNE. Some regional indicators, as we find in the study, would probably also fit within the permitted range. For instance, the near vowel might be considered standard in both its Manchester and Sheffield variant, even though the two variants clearly differ. Some other vowels, on the other hand, may not vary in the same way. For instance, a speaker with a retracted bath vowel may be considered standard, but no longer Northern, whereas a speaker with a monopthongised face may be seen as northern, but no longer standard.

Liverpool and Newcastle systematically depart from any possible description of GNE. Liverpool accent shows robust local features, including systematic fronting of fool, and lowering of letter. Note that both these features may not be entirely localized to Liverpool, based on previous literature. Sources report fool-fronting in working class Manchester speech (Baranowski and Turton, 2015), whereas an open quality of the letter vowel is, in fact, one of the most stereotyped features of Manchester speech. The fact that, in our data we find these two features to be markers of Liverpool, rather than Manchester, might suggest that the two features carry different social meaning in the two cities. Among other possibilities, they may be more stigmatized in Manchester than in Liverpool, such that more standard Manchester speakers avoid them. Note that Manchester speakers in our sample avoid letter lowering. If anything, they have a raised letter vowel compared to other cities. More generally, Liverpool speakers are also likely to differ from other Northern speakers in their attitude toward local features. Although we are not aware of systematic across-city comparisons in this respect, Juskan (2018) presents qualitative data on the attitudes of Liverpool speakers toward their own accent. Some of them explicitly mention the distinctness of Liverpool speech within the UK, and comment on local identity and local pride. A strong sense of local identity is likely to make an accent more resistant to leveling, such that many speakers hold on to at least some regional features. This possibility is consistent with our results. Not only is Liverpool clearly distinct from other cities, but it also shows features that are potentially stigmatized elsewhere in the North (fool-fronting). We also find evidence of Liverpool accent preserving its own unique dialect features. For instance, the median vowel formant measurements for nurse and square in our data are consistent with there being a nurse-square merger in Liverpool, as previously described for this city. Previous research also shows that this feature has relatively low local social prestige (Watson and Clark, 2013), but it resists leveling nonetheless.

Newcastle speech, as represented in our sample, is also distinct, but not because local variants featuring heavily. On the contrary, the Newcastle speakers seem closer to the Southern British standard than the Northern one. One of the salient parameters of variation, in this respect, is that many of the Newcastle speakers had a robust, phonemic-like foot-strut distinction (this is true for half of the Newcastle speakers in this corpus, as analyzed in Strycharczuk et al., 2019). This finding is similar to the results from Halfacre and Khattab (2019), who report a foot-strut split in privately educated speakers from Newcastle. The second most prominent feature of Newcastle speech, a fronted offglide of price, is also a feature of standard speech. We also note from Figure 7 that Newcastle is the only city in our sample with a diphthongal pronunciation of square, which is also typical of SSBE. Meanwhile, the representative vowel charts do not contain any traditional Newcastle vowel features, such as centring diphthongs in face and goat. It is not obvious why standard Newcastle speech should be, in a sense, “less northern,” than the standard speech of Manchester or Leeds speakers. We can speculate that the social status of the local accent in Newcastle is different than in Manchester or Leeds, such that more standard speakers may avoid blending local features into their speech. Negative attitudes toward traditional accent in Newcastle are mentioned in the context of dialect leveling in this city, as observed by Watt (2002). A related hypothesis is that a raised strut vowel is evaluated differently in Newcastle than in other northern UK cities, hence it is not incorporated into the standard. It is also relevant to consider the proximity of Newcastle to the Scottish border. Since Scottish English does not have a foot-strut split, dialect contact might serve to reinforce the split in neighboring varieties.

Throughout the discussion, we have made references to social meaning in our proposed interpretation of the data. We have set out hypotheses about how specific vowel features may be evaluated, and how such evaluations might differ across the North. Perceptual research is necessary to provide a systematic description of General Northern English. Ultimately, standard speech is defined by what listeners perceive as standard, although it is instructive to see how individuals may deviate from that in production, whether or not consciously. In this context, our research not so much settles all the questions surrounding General Northern English, as it tells us where to look further. Our key contribution is identifying the features that are the loci of systematic regional variation, and features that are not. Further research can determine the relationship between this observed variation and the social perception of standard speech in the North.

In order to identify the features that contribute to differentiate regional accents, we have proposed a novel method, based on random forest classification. This method can be extended to comparing any types of groups that may be of sociolinguistic interest. It can also be extended to include additional features, such as consonantal features, and potentially also to categorical variables. An explicit method for feature selection could be a valuable tool in sociolinguistics, informing researchers' choices of what to study. Currently, the feature choices on the part of sociolinguists are not always overtly motivated. Oftentimes, they are simply the features that researchers notice. However, just like any human listeners, linguists can be biased in their perception, paying special attention to features they know about from previous literature, to features that are marked, and to phonetic differences that are big. One unfortunate outcome of this situation is that instances of small but systematic variation can be systematically missed. The tool we have developed is particularly good at identifying such variation, and as such, it can inform research decisions. Due to its success with identifying regional indicators, the method may have also applications in forensic contexts, such as accent profiling.

We developed the method specifically to maximize the returns from using a relatively small speaker sample. From a computational perspective, our sample (*N* = 105) is indeed small. However, it is a fairly standard number of speakers for a study in speech variation. The practicalities of working with speech seriously limit the amount of data we can presently collect and process. The long-term goal for speech variation studies is to scale up the amount of speech data from different varieties, potentially by pooling different corpora. Such work is already under way (e.g., Stuart-Smith et al., 2020), although we are still some way away from having rich large-scale spoken English corpora with good geographical coverage. In the meantime, trying to mitigate against the limitations of existing resources allows us to continue documenting speech variation, improving the methods as we go along.

## 5. Conclusion

In this study, we used random-forest based classification to quantify the mutual levels of similarity of vowel systems in different accents. Our interest was in evaluating the hypothesis that dialect leveling in middle-class Northern English speakers has led to convergence toward a pan-regional General Northern English. We do find some evidence of such convergence, although some accents cluster in this respect (Manchester, Leeds, Sheffield), whereas others remain more distinct (Liverpool, Newcastle). Our proposed interpretation of this geographical variation relies on regional variance in language attitude, and differences in the perception of local dialect prestige and local pride. Furthermore, while some traditional accent features may be recessive, most speakers in our sample can still be reliably localized to their particular city. This is often cued by less described, but nevertheless systematic vowel features. This finding is consistent with the prediction that dialect-leveling predominantly targets marked regional features. However, it also highlights that we need to re-evaluate the relevant parameters for variation when updating dialect descriptions. Our study contributes a method for doing that, which combines the benefits of computational approaches (an explicit computational procedure) with being phonetically interpretable, which in turn, bridges our findings with more traditional variationist work.

## Data Availability Statement

The data and code used in this study are available at https://osf.io/vtp79/.

## Author Contributions

This study was initiated by PS. The data were extracted and prepared by GB and AL, using bespoke tools designed by GB for forced alignment. PS and ML-I collaborated on the conception of the analysis. The analysis was designed and executed by ML-I. PS wrote the manuscript, which was read, revised and approved by all authors.

## Conflict of Interest

The authors declare that the research was conducted in the absence of any commercial or financial relationships that could be construed as a potential conflict of interest.

## Footnotes

^1^GSCE stands for General Certificate of Secondary Education. It is awarded based on individual subject exams generally taken at age 16. An A(Advanced)-level is a further qualification, also awarded based on subject-specific exam results. This qualification is not obligatory, and it also serves as University entry exam.
